# Relationship between Professional Training of Dentists and Outpatient Clinical Production

**DOI:** 10.1155/2022/5365363

**Published:** 2022-03-20

**Authors:** André Scolare Bueno, Roger Keller Celeste

**Affiliations:** Department of Preventive and Social Dentistry, School of Dentistry, Federal University of Rio Grande do Sul, Porto Alegre, Brazil

## Abstract

**Background:**

The aim was to evaluate the association between the professional training of dentists and their outpatient production (OP) of clinical and collective/preventive procedures and the total number of procedures registered in a health information system.

**Methods:**

It included all 19,947 primary dental care units participating in the Program for Improvement of Access and Quality of Primary Care (PMAQ-AB 2nd cycle) and the number of clinical procedures (CP), collective/preventive procedures (PP), and total procedures (TP) registered in the ambulatory information system between November 2013 and July 2014 for each participant oral health team. The outcome was being above the national median of procedures. The main variables related to training were the dentists specialising in family health, the level of training, and participation in permanent education. Effect estimates were calculated by multiple logistic regression.

**Results:**

In the final model, controlled by contextual factor work process, family health specialists had higher chances (odds ratio (OR) = 1.13, 95% CI: 1.00; 1.27) of producing above the national median of CP than nonspecialists, OR = 1.06 (0.96; 1.18) for PP and OR = 1.17 (1.08; 1.27) for TP. Dentists taking permanent education had higher chances than those not taking it of producing above the national median for CP, PP, and TT, respectively, with OR = 1.40 (1.20; 1.62), OR = 1.24 (1.09; 1.40), and OR = 1.28 (1.18; 1.39).

**Conclusion:**

Training in family health performs more procedures in primary care settings than those without training. However, this OP is influenced by variables related to the municipality and the work process, especially for PP. If the highest production observed is a consequence of training, then public health managers can not only encourage training policies such as permanent education policies to expand the use of services.

## 1. Introduction

Healthcare assessment is needed to evaluate if health policy has achieved its goals of transforming a situation towards a more desirable one [1]. The outpatient production of health teams, classified as process evaluation, is directly related to the healthcare system performance and represents the use of services (realised access) [2, 3]. Outpatient production of clinical procedures constitutes an essential way of evaluating healthcare services, representing its interaction with the user [4]. The determinants of use health services and production of clinical procedures have been named as follows: (a) the health needs of users and their characteristics, (b) form of organisation of services, (c) health system policy, and (d) characteristics of health service providers. Health professionals define the type and demand of resources consumed to solve the health problems of patients [5].

In Brazil, the outpatient production of oral health teams (OHT) is processed by municipal and state managers through the Outpatient Information System of the Unified Health System (in Portuguese, Sistema de Informação Ambulatorial do Sistema Único de Saúde, acronym SIA/SUS) [6]. That production can be influenced by the infrastructure of healthcare facilities [7], by its oral healthcare network and care model [8–10], by the working process of the teams [11], through self-assessment [12], or by the training of dentists [13]. Araújo et al. highlight that professionals trained in the Brazilian Unified Health System (SUS) tend to know it better. Investments in training are of paramount importance for changes in the healthcare model, specifically in Primary Health Care (aka Family Health Strategy in Brazil, acronym FHS) [14].

The Brazilian National Health System has a legal responsibility in managing the training of healthcare personnel [15]. Despite that, no study has yet investigated the relationship between the training offered to dentists and their outpatient production. Recently, a study described the association between the variety of procedures according to different dentists' profile [16], but another study showed that the production of SUS is different according to geographical regions [17]. Due to local differences in the context of each catchment area of the OHTs, it would be expected that dentists with residence/specialisation in family health or public health may have a greater and more diversified number of procedures. Those professionals were trained to know the system [14] and also to include dental assistants as part of the team, different from the traditional biomedical model where dentist usually works alone. Dental assistants are known to have clinical competencies to increase productivity [18, 19].

Therefore, this study was aimed at evaluating the association between the professional training of dentists and their outpatient production of clinical and collective/preventive procedures and the total number of procedures registered in a health information system.

## 2. Methods

This study included data from a census of all primary dental care units in Brazil during 2013 and 2014. Approximately 24,533 dental care units across the country and 19,946 (81.3% response rate) adhered to the 2^nd^ cycle of the Program for Improvement of Access and Quality of Primary Health Care (in Portugues called Programa de Melhoria do Acesso e Qualitade da Atenção Básica, acronym PMAQ-AB). Among the participant units, 17,259 OHT dentists comprised the current sample. All data is available at https://aps.saude.gov.br/ape/pmaq/ciclo2/. Data from PMAQ-AB 2nd cycle was linked with data from the outpatient system (SIA-SUS) at primary dental care units' level from November 2013 to July 2014. All information is freely available at https://datasus.saude.gov.br/.

### 2.1. Outcome Variables

The three outcome variables were the number of clinical and collective/preventive procedures and total procedures approved in SIA-SUS (see supplemental file for codes grouped into each category of procedures (available here)). Total procedures include clinical, preventive, and other procedures, such as home visits, nonplanned consultations (e.g., urgency), health surveillance, and nondental procedures (e.g., hypertension measurements taken by dentists). The statistical program R was used for data extraction and processing using the “microdatasus” and “tidyverse,” respectively [20, 21]. The “month_end” argument was extended to 3 months later due to its correspondence to the time interval in which the municipality can still make changes in the system [6]. The production was filtered by the dentist occupation codes, according to the Brazilian Classification of Occupations - CBO2002 [22]. The grouping was carried out by the “CNES” variable, the unique primary care unit identifier. In addition, the production of units that had more than one dentist (9.1%) was divided by the corresponding number of professionals. Original variables were counts of procedures at unit level; then, they were dichotomised in the median for further statistical analysis.

### 2.2. Main Predictor Variables

The three main variables related to professional training were obtained from Module VI of the PMAQ-AB 2nd cycle. Two variables were created from the question “VI_3_2 - Which of these training process certifications do you possess?”. The first corresponds to training with an emphasis on family health, being classified as follows: (a) “yes, in family health”—including professionals who answered “completed” to at least one of the questions referring to having specialisation, residence, master, or doctorate in family health/public health/collective health; (b) “yes”—for those who answered “concluded” for at least one of the variables of having a postgraduate degree different from family health; and (c) “no”—includes other professionals without a postgraduate degree. The second variable separated professionals who answered “completed” only for “Lato Sensu” or “Stricto Sensu,” for both “Lato and Stricto Sensu” and “none.” In the third variable, comprising the participation of permanent education, we considered the question “VI_6_1_11 - Does not participate in any form of permanent education”.

### 2.3. Covariates

In our study, we considered as potential confounding factors the following covariates: geographical region, city size, and percentage of residents in urban areas. All covariates were obtained from the 2010 census. The percentage of coverage of the FHS and OHT was obtained from the raw database of the E-Manager (E-Gestor). The variables related to the dentists working at OHT were obtained from the PMAQ-AB database: “VI_2_4 - How long have you worked in this primary care team?”; “VI_5_5 - Do you have an incentive, bonus, financial reward for performance?”; “VI_10_1_1 - Yes, from the DSC (Dental Specialties Centers).”; “VI_10_1_6 – No (Concerning Institutional Support)”; and “VI_7_5_2 - Which instrument/source was used? (Referring to Self-Assessment)”. The inverse result of the variable “VI_10_1_6” was used to facilitate interpretation. The question “VI_7_5_2” was grouped with the answers “AMAQ” and “AMQ,” other self-assessments, and self-assessment not included. Still, the question VI_2_4 allowed categorisation of professionals in “0-2 years,” “3-6 years,” and “more than 6 years” of work at the OHT.

In addition to those variables, the number of dental X-ray, dental equipment, light curing unit, and dental ultrasound was collected from Module V of the PMAQ-AB, and a continuous variable was created with the sum of the equipment.

We theoretically considered as potential mediators of the relationship between the outcome and the main variables the question “VI_6_7 - How often does the unit host students, professors and/or researchers in teaching, research and/or community extension activities?”. If the answer had weekly frequency, we classified it as “yes” and the rest as “no.” Moreover, variables related to the organisation of the dentists' schedule, “VI_13_1 - The team's clinical care schedule ensures” and “VI_13_6 - How are dental appointments scheduled at the Health Unit?”, were grouped into “fixed days,” if presented a specific day for scheduling.

The hypothesis of these variables being mediators stems from the assumption that professionals with FH training would be more likely to develop teaching, research, and extension in partnerships with educational institutions, intending structural changes in their health unit towards a scheduling format and guaranteed access to SUS.

### 2.4. Statistical Analysis

Variables were described using mean and standard deviation for continuous variables and absolute and relative frequency for categorical variables. Bivariate analyses of the outcome with the other covariates were performed using the Kruskal-Wallis test with a significance level of 5%.

Effect estimates were calculated by multiple regression using the binomial model with a logit link function (logistic model), with the outcome being dichotomised by its median. The option to dichotomise was due to the asymmetric nature of the outcome data with several influential outliers and substantial standard deviations, as observed in a preliminary analysis.

Three models were tested in the regression analysis. The crude model includes only the main variables. Model 1 includes each main variable independently and the confounding variables described previously. Model 2 includes variables in model 1 (main predictors not adjusted by each other) and those that theoretically could mediate the effect of main variables on outpatient production. Finally, to test if the associations in each main variable differed by macroregion, we included interaction terms in regression models. Data were analysed using the R version 4.0.3 statistical software.

## 3. Results

It was observed that of the 19,947 participants in the external evaluation of the PMAQ AB 2nd cycle, 17,259 (86.4%) were OHT dentists that composed the analytical sample. All 17,259 units were units registered in the CNES database of healthcare facilities, and 81.4% of them had only one dentist, showing that in most observations, the unit's outpatient production refers to the dentist who answered to the interviewed professional at PMAQ-AB and is not influenced by the work of another professional.

According to Table 1, 57.1% of the interviewed dentists had additional training, mostly at graduation level. Specialisation in family health represents 29.5% of the sample, and those professionals produce, on average, a greater number of collective/preventive procedures and total production. Also, dentists who participate in permanent education have a higher average of procedures in all outcome variables. The mean of the three outcome variables was associated with all main variables.

In the multiple logistic regression analysis, it was observed that specialists in family health presented had a greater outpatient production for clinical procedures (OR = 1.44; 95% CI: 1.29-1.61), collective/preventive procedures (OR = 1.38; 95% CI: 1.26-1.52), and total procedures (OR = 1.51; 95% CI: 1.40; 1.62). The same pattern was reported for joint training at Lato and Stricto sensu and participating in permanent education, resulting in raw models with *p* < 0.01.

Tables 2, 3, and 4 present the results of the adjusted models for each outcome variable. In the adjusted model 1, we observed that, after controlling for factors at the city level where the dentist works and the OTH work process, professionals with training in family health are the only ones who maintain a significant difference concerning the measurement of clinical procedures (*p* = 0.04) and the total number of procedures (*p* < 0.01). In addition, we emphasise that professionals who participate in permanent education produce, on average, a greater number of clinical and collective/preventive procedures and total procedures, both with *p* < 0.01.

The adjusted model 2 is controlled by variables in model 1 and potential mediators. Model 2 had a higher BIC than model 1, showing that it has a worse fit. The magnitude of main associations remained unchanged, meaning that those variables may contain substantial measurement error or they may not be mediators. The interaction results by macroregions (effect modification of “area of postgraduate course”) were not statistically significant at a level of 5% and varied around OR = 1, sometimes in an uninterpretable way.

## 4. Discussion

The study shows that the outpatient production of specialists in public health or family health is larger than other specialists, but this result is partially influenced by the municipality context factors and work process. Regarding collective/preventive procedures, we found a smaller and statistically not significant difference in the adjusted model. However, for clinical procedures and total procedures, this speciality was the only one that presented a significant result. When explaining such a weak and nonsignificant association, one should consider that collective procedures include dental health education activities (see supplemental file) conducted by dental hygienists at a school environment [23]. Although more collective procedures have been associated with higher coverage of FHS [10], such increase is likely due to the presence of dental auxiliaries. Therefore, collective procedures may not be affected by training dentists who work mostly in clinical setting. Still, professionals who take part in permanent education perform more procedures.

The present study has limitations and strengths. Outpatient production can be under- or overreported in information systems [8–10]. Nonetheless, no study has evaluated the reliability and validity of SIA-SUS, unlike other health information systems [24]. Barros and Chaves suggest that traditional dental procedures, such as the number of tooth extractions and restorations, show higher validity and reliability in their records [25]. A relevant limitation is that motivated professionals, who are predisposed to greater production, may also carry out training courses and permanent education, resulting in bias. As for strengths, we highlight the absence of selection bias, as the analyses used a census of health teams. Additionally, the information in the PMAQ-AB database was obtained *in locus*, minimising measurement errors.

The influence of participation in continuing education activities shows the relevance of this type of initiative, where the clinical topics in those education activities are present in the daily practice. Some studies [26, 27] highlight that there is still conceptual confusion among health professionals and managers between the terms permanent education and continuing education, leading to a different interpretation of the results. In our study, the variable is a compilation of a group of questions that described only continuing education activities. Maciel et al. [26] reported the lack of studies that analyse permanent education in health linked to the work process of dental surgeons who work in the Family Health Strategy. We identified a couple of studies investigating the association between dentists' profile and a validated roster of 23 self-reported clinical procedures [16, 28] included in the current study. They found that continuing professional training was associated with a larger variety of procedures but were not able to define the amount of each one. Our findings contribute to confirm the effect of permanent education in the Primary Health Care (PHC) service.

Our data show that professionals with a Lato Sensu degree have greater production, thus expanding access to populations with large unmet needs. In Brazil, postgraduate courses have advanced the qualification of primary care professionals. By mid-June 2020, the Open University of SUS (Universidade Aberta do SUS, UNA-SUS) had already trained more than 90,000 professionals in specialisation courses, about 77,000 of them in family health [29], which means a big step forward in a possible change in the healthcare model. However, at the undergraduate level, the growing and unrestrained increase in new dental schools, mainly in the private sector, has contributed to increasing regional asymmetries in the supply of dentists and fostering an educational logic focused on the private market [30, 31].

## 5. Conclusions

In conclusion, it was shown that dentists participating in the PMAQ-AB 2nd cycle with training in public health or family health performed more clinical procedures. However, other factors are essential in addition to professional training, especially in producing collective/preventive procedures. Future studies can confirm the current findings and investigate the processes that apparently lead professionals with broad-based education to have greater production. If the higher production observed is a consequence of training, then public health managers can encourage training policies and permanent education policies as a way to expand the use of services.

## Figures and Tables

**Table 1:** Description of average counts of procedures at primary dental care unit according to professional training of dentists participating from November 2013 to July 2014 in the SIA-SUS.

	*N* (%)	Clinical procedures (average; ±SD)	*p* value	Collective/preventive procedures (average; ±SD)	*p* value	Total procedures (average; ±SD)	*p* value
Area of postgraduate course^∗^			<0.01		<0.01		<0.01
No	7416 (43.0)	224.7; 1.857.8		210.7; 1.404.1		563.9; 2.821.3	
Yes, any other area	4756 (27.6)	238.5; 2.051.6		217.5; 1.297.5		564.9; 2.783.1	
Yes, in family health/public health	5087 (29.5)	233.9; 1.648.4		265.4; 1.553.9		645.1; 2.933.9	

Type of postgraduate course			<0.01		<0.01		<0.01
None	7416 (43.0)	224.7; 1.857.8		210.7; 1.404.1		563.9; 2.821.3	
Lato sensu	8655 (50.1)	242.7; 1.945.6		239.2; 1.374.1		615.2; 2.952.8	
Stricto sensu	253 (1.47)	128.1; 102.5		128.3; 189.4		343.6; 463.0	
Lato sensu and Stricto sensu	935 (5.42)	204.6; 1.069.9		301.8; 2.052.8		595.0; 2.336.4	

Permanent education?			<0.01		<0.01		<0.01
No	3112 (18.0)	195.9; 1.260.1		171.8; 1.066.1		478.3; 2.218.1	
Yes	14147 (82.0)	239.0; 1.962.5		241.2; 1.489.1		612.3; 2.964.3	

^∗^Specialisation, residence, master, or doctorate. SD = standard deviation.

**Table 2:** Crude and adjusted odds ratio (OR) from logistic regression models (95% confidence interval) for the association of being above the median outpatient production of *clinical procedures* by the oral health teams participating in the second cycle of PMAQ-AB.

	Crude model		Adjusted model 1		Adjusted model 2	
	OR (95% CI)	*p* value	OR (95% CI)	*p* value	OR (95% CI)	*p* value
Area of postgraduate course^∗^						
No	1	<0.01	1	0.04	1	0.04
Yes, any other area	1.02 (0.90-1.15)		0.97 (0.86-1.10)		0.97 (0.86-1.10)	
Yes, in family health	1.44 (1.29-1.61)		1.13 (1.00-1.27)		1.13 (1.00-1.27)	
BIC	12341		12178		12210	

Type of postgraduate course						
None	1	<0.01	1	0.05	1	0.05
Lato sensu	1.24 (1.12-1.37)		1.07 (0.96-1.18)		1.06 (0.96-1.18)	
Stricto sensu	0.58 (0.35-0.97)		0.55 (0.33-0.92)		0.55 (0.32-0.92)	
Lato sensu and Stricto sensu	1.37 (1.12-1.68)		1.06 (0.86-1.31)		1.06 (0.86-1.31)	
BIC	12371		12186		12217	

Takes part in permanent education?						
No	1	<0.01	1	<0.01	1	<0.01
Yes	1.65 (1.43-1.90)		1.40 (1.20-1.62)		1.38 (1.19-1.60)	
BIC	12327		12153		12187	

Note: variables in the first column are not adjusted by one another. BIC: Bayesian information criteria. Model 1-adjusted by potential confounding factor: macroregion + population size + urban area (%) + FHS and OHT coverage (%) + team time + performance incentive + institutional support + DSC support + self-assessment. Model 2-adjusted by model 1 and mediation factors: receiving students + guaranteed scheduling + form of scheduling appointments.

**Table 3:** Crude and adjusted odds ratio (OR) from logistic regression models (95% confidence interval) for the association of being above the median outpatient production of *collective/preventive procedures* by the oral health teams participating in the second cycle of PMAQ-AB.

	Crude model		Adjusted model 1		Adjusted model 2	
	OR (95% CI)	*p* value	OR (95% CI)	*p* value	OR (95% CI)	*p* value
Area of postgraduate course^∗^						
No	1	<0.01	1	0.29	1	0.29
Yes, any other area	1.05 (0.95-1.17)		0.98 (0.88-1.08)		0.98 (0.88-1.08)	
Yes, in family health	1.38 (1.26-1.52)		1.06 (0.96-1.18)		1.06 (0.96-1.18)	
BIC	15612		15135		15174	

Type of postgraduate course						
None	1	<0.01	1	0.55	1	0.56
Lato sensu	1.23 (1.13-1.33)		1.03 (0.94-1.12)		1.03 (0.94-1.12)	
Stricto sensu	0.77 (0.53-1.13)		0.79 (0.54-1.17)		0.79 (0.54-1.17)	
Lato sensu and Stricto sensu	1.30 (1.09-1.55)		0.99 (0.83-1.20)		0.99 (0.83-1.20)	
BIC	15642		15145		15185	

Takes part in permanent education?						
No	1	<0.01	1	<0.01	1	<0.01
Yes	1.53 (1.36-1.71)		1.24 (1.09-1.40)		1.24 (1.09-1.40)	
BIC	15596		15115		15155	

Note: variables in the first column are not adjusted by one another. BIC: Bayesian information criteria. Model 1-adjusted by potential confounding factor: macroregion + population size + urban area (%) + FHS and OHT coverage (%) + team time + performance incentive + institutional support + DSC support + self-assessment. Model 2-adjusted by model 1 and mediation factors: receiving students + guaranteed scheduling + form of scheduling appointments.

**Table 4:** Crude and adjusted odds ratio (OR) from logistic regression models (95% confidence interval) for the association of being above the median outpatient production of *total number of procedures* of the oral health teams participating in the second cycle of PMAQ-AB.

	Crude model		Adjusted model 1		Adjusted model 2	
	OR (95% CI)	*p* value	OR (95% CI)	*p* value	OR (95% CI)	*p* value
Area of postgraduate course^∗^						
No	1	<0.01	1	<0.01	1	<0.01
Yes, any other area	1.03 (0.96-1.11)		0.97 (0.90-1.05)		0.97 (0.90-1.05)	
Yes, in family health	1.51 (1.40-1.62)		1.17 (1.08-1.27)		1.18 (1.09-1.28)	
BIC	23811		22803		22819	

Type of postgraduate course						
None	1	<0.01	1	0.04	1	0.04
Lato sensu	1.25 (1.18-1.33)		1.07 (1.00-1.14)		1.07 (1.00-1.14)	
Stricto sensu	0.80 (0.62-1.03)		0.80 (0.61-1.04)		0.80 (0.61-1.04)	
Lato sensu and Stricto sensu	1.43 (1.24-1.64)		1.09 (0.94-1.26)		1.09 (0.94-1.26)	
BIC	23893		22827		22844	

Takes part in permanent education?						
No	1	<0.01	1	<0.01	1	<0.01
Yes	1.62 (1.50-1.75)		1.28 (1.18-1.39)		1.29 (1.18-1.40)	
BIC	23797		22782		22798	

Note: variables in the first column are not adjusted by one another. BIC: Bayesian information criteria. Model 1-adjusted by potential confounding factor: macroregion + population size + urban area (%) + FHS and OHT coverage (%) + team time + performance incentive + institutional support + DSC support + self-assessment. Model 2-adjusted by model 1 and mediation factors: receiving students + guaranteed scheduling + form of scheduling appointments.

## Data Availability

All information used in the current manuscript is freely available at Health Information Systems. PMAQ-AB microdata is available at the website of the Ministry of Health of Brazil: https://aps.saude.gov.br/ape/pmaq.

## Supplementary Materials

Supplementary Materials The supplemental file contains the codes of procedures that were grouped into each analytical category.
