# Supplementary material for: Relationship between Professional Training of Dentists and Outpatient Clinical Production
Source: Biomed Res Int. 2022 Mar 20;2022:5365363. doi: 10.1155/2022/5365363 (PMC8958072; doi:10.1155/2022/5365363)
Supplement: Supplementary Materials — The supplemental file contains the codes of procedures that were grouped into each analytical category. [file 5365363.f1.pdf]

Supplementary table

Classification of procedures approved in SIA-SUS performed by Dental Surgeons, following the Brazilian Classification of Occupations - CBO2002. (Codes:"223204","223208","223280","223284","223212","223216","223220","223224","223228","223276","223288","223232","223236","223240","223244","223248","223252","223256","223260","223264","223268","223272","2232B1","223293")

Classification	
Clinical Procedures	Absolute and Relative Frequency (%)
Operative Dentistry	**15.034.170 (16,47)**
RESTAURAÇÃO DE DENTE PERMANENTE POSTERIOR	8.000.957 (53,22)
RESTAURAÇÃO DE DENTE PERMANENTE ANTERIOR COM RESINA COMPOSTA	3.069.837 (20,42)
RESTAURAÇÃO DE DENTE DECÍDUO	2.055.461 (13,67)
CAPEAMENTO PULPAR	1.897.271 (12,62)
TRATAMENTO DE NEVRALGIAS FACIAIS	10.644 (0,07)
Periodontics	**12.677.762 (13,89)**
RASPAGEM ALISAMENTO E POLIMENTO SUPRAGENGIVAIS (POR SEXTANTE)	9.268.243 (73,11)
RASPAGEM ALISAMENTO SUBGENGIVAIS (POR SEXTANTE)	3.104.752 (24,49)
RASPAGEM CORONO-RADICULAR (POR SEXTANTE)	286.636 (2,26)
GENGIVECTOMIA (POR SEXTANTE)	7.146 (0,06)
ODONTOSECÇÃO / RADILECTOMIA / TUNELIZAÇÃO	5.481 (0,04)
TRATAMENTO CIRÚRGICO PERIODONTAL (POR SEXTANTE)	3.187 (0,03)
GENGIVOPLASTIA (POR SEXTANTE)	1.916 (0,02)
ENXERTO GENGIVAL	401 (0,00)
Oral Surgery	**5.494.875 (6,02)**
EXODONTIA DE DENTE PERMANENTE	2.427.775 (44,18)
EXODONTIA DE DENTE DECÍDUO	1.479.260 (26,92)
EXCISÃO E/OU SUTURA SIMPLES DE PEQUENAS LESÕES / FERIMENTOS DE PELE / ANEXOS E MUCOSA	594.750 (10,82)
TRATAMENTO CIRÚRGICO DE HEMORRAGIA BUCO-DENTAL	344.887 (6,28)
ULOTOMIA/ULECTOMIA	341.507 (6,22)
TRATAMENTO DE ALVEOLITE	149.833 (2,73)
DRENAGEM DE ABSCESSO	76.210 (1,39)
CURETAGEM PERIAPICAL	28.316 (0,52)
EXODONTIA MÚLTIPLA COM ALVEOLOPLASTIA POR SEXTANTE	10.534 (0,19)
EXCISÃO E SUTURA DE LESÃO NA BOCA	8.857 (0,16)
REMOÇÃO DE DENTE RETIDO (INCLUSO / IMPACTADO)	8.276 (0,15)
FRENÉCTOMIA	5.825 (0,11)
GLOSSORRAFIA	5.146 (0,09)
DRENAGEM DE ABSCESSO DA BOCA E ANEXOS	4.276 (0,08)
EXCISAO DE LESAO E/OU SUTURA DE FERIMENTO DA PELE ANEXOS E MUCOSA	3.575 (0,07)
CORREÇÃO DE IRREGULARIDADES DE REBORDO ALVEOLAR	2.283 (0,04)
REMOÇÃO DE TORUS E EXOSTOSES	1.151 (0,02)
RETIRADA DE MATERIAL DE SÍNTESE ÓSSEA / DENTÁRIA	668 (0,01)
APROFUNDAMENTO DE VESTÍBULO ORAL (POR SEXTANTE)	302 (0,01)
CORREÇÃO DE BRIDAS MUSCULARES	287 (0,01)
MARSUPIALIZAÇÃO DE CISTOS E PSEUDOCISTOS	253 (0,00)
REIMPLANTE E TRANSPLANTE DENTAL (POR ELEMENTO)	205 (0,00)
CONTENÇÃO DE DENTES POR SPLINTAGEM	120 (0,00)
BIÓPSIA DOS TECIDOS MOLES DA BOCA	109 (0,00)
EXERESE DE TUMOR DE PELE E ANEXOS / CISTO SEBACEO / LIPOMA	91 (0,00)
ENXERTO ÓSSEO DE ÁREA DOADORA INTRABUCAL	90 (0,00)
EXERESE DE CISTO ODONTOGÊNICO E NÃO-ODONTOGÊNICO	76 (0,00)
CORREÇÃO DE TUBEROSIDADE DO MAXILAR	70 (0,00)
EXCISÃO DE CÁLCULO DE GLÂNDULA SALIVAR	37 (0,00)
OSTEOTOMIA DAS FRATURAS ALVEOLO-DENTÁRIAS	20 (0,00)
TRATAMENTO CIRÚRGICO DE FÍSTULA INTRA / EXTRAORAL	19 (0,00)
APICECTOMIA COM OU SEM OBTURAÇÃO RETRÓGRADA	16 (0,00)
INCISAO E DRENAGEM DE ABSCESSO	16 (0,00)
RECONSTRUÇÃO PARCIAL DO LÁBIO TRAUMATIZADO	13 (0,00)
REDUÇÃO DE FRATURA ALVEOLO-DENTÁRIA SEM OSTEOSSÍNTESE	5 (0,00)
TRATAMENTO CIRÚRGICO PARA TRACIONAMENTO DENTAL	5 (0,00)
REDUÇÃO DE LUXAÇÃO TÊMPORO-MANDIBULAR	3 (0,00)
TAMPONAMENTO NASAL ANTERIOR E/OU POSTERIOR	3 (0,00)
BIÓPSIA DE GLÂNDULA SALIVAR	2 (0,00)
EXCISÃO DE RÂNULA OU FENÔMENO DE RETENÇÃO SALIVAR	2 (0,00)
BIÓPSIA DE OSSO DO CRÂNIO E DA FACE	1 (0,00)
RETIRADA DE CORPO ESTRANHO DOS OSSOS DA FACE	1 (0,00)
Endodontics	**1.872.410 (2,05)**
ACESSO A POLPA DENTARIA E MEDICACAO (POR DENTE)	1.028.629 (54,94)
CURATIVO DE DEMORA C/ OU S/ PREPARO BIOMECANICO	407.808 (21,78)
PULPOTOMIA DENTÁRIA	344.054 (18,37)
TRATAMENTO ENDODÔNTICO DE DENTE DECÍDUO	30.385 (1,62)
TRATAMENTO ENDODÔNTICO DE DENTE PERMANENTE BIRRADICULAR	16.366 (0,87)
TRATAMENTO ENDODÔNTICO DE DENTE PERMANENTE ANTERIOR	15.904 (0,85)
TRATAMENTO ENDODÔNTICO DE DENTE PERMANENTE COM TRÊS OU MAIS RAÍZES	13.792 (0,74)
SELAMENTO DE PERFURAÇÃO RADICULAR	5.650 (0,30)
RETRATAMENTO ENDODÔNTICO EM DENTE PERMANENTE BI-RADICULAR	4.392 (0,23)
RETRATAMENTO ENDODÔNTICO EM DENTE PERMANENTE UNI-RADICULAR	3.671 (0,20)
RETRATAMENTO ENDODÔNTICO EM DENTE PERMANENTE COM 3 OU MAIS RAÍZES	1.759 (0,09)
Prosthodontics and Rehabilitation	**422.622 (0,46)**

AJUSTE OCLUSAL	232.178 (54,94)
MOLDAGEM DENTO-GENGIVAL P/ CONSTRUCAO DE PROTESE DENTARIA	81.693 (19,33)
INSTALAÇÃO DE PRÓTESE DENTÁRIA	36.690 (8,68)
ADAPTAÇÃO DE PRÓTESE DENTÁRIA	18.465 (4,37)
PROTESE TOTAL MAXILAR	14.167 (3,35)
PROTESE TOTAL MANDIBULAR	10.648 (2,52)
REEMBASAMENTO E CONSERTO DE PROTESE DENTARIA	7.135 (1,69)
PROTESE PARCIAL MANDIBULAR REMOVIVEL	6.990 (1,65)
MANUTENCAO PERIODICA DE PROTESE BUCO-MAXILO-FACIAL	5.407 (1,28)
PROTESE PARCIAL MAXILAR REMOVIVEL	4.980 (1,18)
CIMENTAÇÃO DE PRÓTESE DENTÁRIA	1.864 (0,44)
PROTESES CORONARIAS / INTRA-RADICULARES FIXAS / ADESIVAS (POR ELEMENTO)	815 (0,19)
COLOCACAO DE PLACA DE MORDIDA	556 (0,13)
MANUTENÇÃO/CONSERTO DE APARELHO ORTODÔNTICO/ORTOPÉDICO	456 (0,11)
COROA PROVISORIA	346 (0,08)
PLANO INCLINADO	107 (0,03)
PRÓTESE MANDIBULAR	38 (0,01)
PRÓTESE PARA GRANDES PERDAS MAXILARES	30 (0,01)
PLACA OCLUSAL	24 (0,01)
APARELHO ORTOPÉDICO E ORTODÔNTICO REMOVÍVEL	19 (0,00)
PROTESE TEMPORARIA	14 (0,00)
Diagnosis	**143.131 (0,16)**
RADIOGRAFIA PERI-APICAL INTERPROXIMAL (BITE-WING)	139.917 (97,75)
RADIOGRAFIA OCLUSAL	1.713 (1,20)
TESTE RÁPIDO PARA SÍFILIS	652 (0,46)
TESTE RÁPIDO PARA DETECÇÃO DE INFECÇÃO PELO HIV	490 (0,34)
RADIOGRAFIA PANORAMICA	193 (0,13)
TRIAGEM OFTALMOLÓGICA	122 (0,09)
TESTE RAPIDO PARA DETECCAO DE HIV NA GESTANTE OU PAI/PARCEIRO	23 (0,02)
TESTE RÁPIDO PARA SÍFILIS NA GESTANTE OU PAI/PARCEIRO	17 (0,01)
RADIOGRAFIA BILATERAL DE ORBITAS (PA + OBLIQUAS + HIRTZ)	1 (0,00)
RADIOGRAFIA DE ARTICULACAO TEMPORO-MANDIBULAR BILATERAL	1 (0,00)
RADIOGRAFIA DE MAXILAR (PA + OBLIQUA)	1 (0,00)
TELERADIOGRAFIA COM TRACADOS E SEM TRACADOS	1 (0,00)
Oral Health Promotion and Preventive Procedures	
Prevention	**35.458.373 (38,84)**
AÇÃO COLETIVA DE ESCOVAÇÃO DENTAL SUPERVISIONADA	12.270.598 (34,61)
AÇÃO COLETIVA DE APLICAÇÃO TÓPICA DE FLÚOR GEL	3.808.854 (10,74)
AÇÃO COLETIVA DE BOCHECHO FLUORADO	3.654.895 (10,31)
APLICAÇÃO TÓPICA DE FLÚOR (INDIVIDUAL POR SESSÃO)	3.414.241 (9,63)
AÇÃO COLETIVA DE EXAME BUCAL COM FINALIDADE EPIDEMIOLÓGICA	3.151.311 (8,89)
SELAMENTO PROVISÓRIO DE CAVIDADE DENTÁRIA	2.955.403 (8,33)
EVIDENCIAÇÃO DE PLACA BACTERIANA	2.318.824 (6,54)
ATIVIDADE EDUCATIVA / ORIENTAÇÃO EM GRUPO NA ATENÇÃO PRIMÁRIA	2.071.834 (5,84)
APLICAÇÃO DE SELANTE (POR DENTE)	1.420.603 (4,01)
APLICAÇÃO DE CARIOSTÁTICO (POR DENTE)	333.604 (0,94)
ABORDAGEM COGNITIVA COMPORTAMENTAL DO FUMANTE (POR ATENDIMENTO / PACIENTE)	40.042 (0,11)
ATIVIDADE EDUCATIVA / ORIENTAÇÃO EM GRUPO NA ATENÇÃO ESPECIALIZADA	17.563 (0,05)
ATIVIDADE EDUCATIVA PARA A POPULAÇÃO	601 (0,00)
Other procedures	
Non-dental procedures performed by Dentists or Non-treatment consultations	**15.434.190 (16,91)**
PRIMEIRA CONSULTA ODONTOLOGICA PROGRAMÁTICA	9.796.068 (63,47)
CONSULTA DE PROFISSIONAIS DE NÍVEL SUPERIOR NA ATENÇÃO PRIMÁRIA (EXCETO MÉDICO)	3.799.748 (24,62)
RETIRADA DE PONTOS DE CIRURGIAS (POR PACIENTE)	1.784.865 (11,56)
AFERIÇÃO DE PRESSÃO ARTERIAL	40.083 (0,26)
CONSULTA DE PROFISSIONAIS DE NIVEL SUPERIOR NA ATENÇÃO ESPECIALIZADA (EXCETO MÉDICO)	13.420 (0,09)
EMISSÃO DE PARECER SOBRE NEXO CAUSAL	6 (0,00)
Urgencies	**3.082.272 (3,38)**
ATENDIMENTO DE URGÊNCIA EM ATENÇÃO BÁSICA	3.075.129 (99,77)
ATENDIMENTO DE URGENCIA EM ATENCAO ESPECIALIZADA	6.592 (0,21)
ATENDIMENTO DE URGENCIA C/ OBSERVACAO ATE 24 HORAS EM ATENCAO ESPECIALIZADA	551 (0,02)
Home Visits	**1.663.780 (1,82)**
CONSULTA/ATENDIMENTO DOMICILIAR	1.476.489 (88,74)
VISITA DOMICILIAR/INSTITUCIONAL POR PROFISSIONAL DE NÍVEL SUPERIOR	187.253 (11,25)
BUSCA ATIVA	34 (0,00)
ASSISTÊNCIA DOMICILIAR POR EQUIPE MULTIPROFISSIONAL.	2 (0,00)
VISITA DOMICILIAR POR PROFISSIONAL DE NÍVEL SUPERIOR	2 (0,00)
Health Surveilance	**66 (0,00)**
INSPEÇÃO DOS ESTABELECIMENTOS SUJEITOS À VIGILÂNCIA SANITÁRIA	39 (59,09)
ATIVIDADES EDUCATIVAS SOBRE A TEMÁTICA DA DENGUE,REALIZADAS PARA A POPULAÇÃO	15 (22,73)
LICENCIAMENTO DOS ESTABELECIMENTOS SUJEITOS À VIGILÂNCIA SANITÁRIA	4 (6,06)
ATENDIMENTO À DENÚNCIAS/RECLAMAÇÕES	3 (4,55)
RECEBIMENTO DE DENÚNCIAS/RECLAMAÇÕES	3 (4,55)
CADASTRO DE ESTABELECIMENTOS SUJEITOS À VIGILÂNCIA SANITÁRIA	1 (1,52)
EXCLUSÃO DE CADASTRO DE ESTABELECIMENTOS SUJEITOS À VIGILÂNCIA SANITÁRIA COM ATIVIDADES ENCERRADA	1 (1,52)
	**91.283.651 (100)**
